# The efficacy and safety of *Danggui-Sayuk-Ga-Osuyu-Saenggang-tang* on Korean patients with cold hypersensitivity in the hands: study protocol for a pilot, double-blind, randomized, placebo-controlled, parallel-group clinical trial

**DOI:** 10.1186/s13063-017-2002-8

**Published:** 2017-06-08

**Authors:** Youme Ko, Ho-Yeon Go, Yoon-Young Cho, Ji-Hye Shin, Tae-Hoon Kim, Dong-Jun Choi, Jin-Moo Lee, Jun-Bok Jang, Yun-Kyung Song, Seong-Gyu Ko, Seung-Ho Sun, Chan-Yong Jeon

**Affiliations:** 1 Department of Korean Preventive Medicine, Graduate School, Kyung Hee University, 26 Kyungheedae-ro, Dongdaemun-gu, Seoul Republic of Korea; 2 Department of Korean Internal Medicine, College of Korean Medicine, Semyung University, 65, Semyeong-ro, Jecheon-si, Chungcheongbuk-do Republic of Korea; 3 Department of Korean Internal Medicine, College of Korean Medicine, Sangji University, 80, Sangjidae-gil, Wonju-si, Gangwon-do 26339 Republic of Korea; 4 Department of Clinical Trial Center, College of Korean Medicine, Kyung Hee University Hospital, 23 Kyungheedae-ro, Dongdaemun-gu, Seoul Republic of Korea; 5 Department of Korean Internal Medicine, College of Korean Medicine, Dongguk University, 814 Siksa-dong, Ilsandong-gu, Goyang-si, Gyeonggi-do Republic of Korea; 6 Department of Korean Gynecology, College of Korean Medicine, Kyung Hee University, 26 Kyungheedae-ro, Dongdaemun-gu, Seoul Republic of Korea; 7 Department of Korean Rehabilitation Medicine, College of Korean Medicine, Gachon University, 1342 Seongnamdae-ro, Sujeong-gu, Gyeonggi-do Republic of Korea; 8 Department of Korean Internal Medicine, College of Korean Medicine, Gachon University, 1342 Seongnamdae-ro, Sujeong-gu, Gyeonggi-do 13120 Republic of Korea

**Keywords:** Herbal medicine, Cold temperature, Cold hypersensitivity, Randomized clinical trial

## Abstract

**Background:**

In recent years, cold hypersensitivity in the hands (CHH) has become a common ailment of women in Korea. It can lead to gynecological problems such as irregular menstruation, miscarriage, and infertility. Traditionally, Korean herbal medicine has been the primary treatment method used to balance thermoregulation in the human body; however, its effectiveness has not been confirmed through systematic study. Thus, in this trial, we will investigate the feasibility of a full randomized clinical trial, *Danggui-Sayuk-Ga-Osuyu-Saenggang-tang* (DSGOST) in Korean women with CHH.

**Methods:**

This study will be a pilot, multicenter, double-blind, randomized, parallel-group, two-arm, placebo-controlled clinical trial. A total of 66 participants will be randomly divided into two groups, a DSGOST treatment group and a placebo control group, in a 1:1 ratio using a web-based randomization system. Each group will take DSGOST or placebo three times daily for 6 weeks. The primary outcome will be measured using Visual Analogue Scale (VAS) scores of CHH. Secondary outcomes will include changes in skin temperature of the hands, Clinical Global Impressions (CGI) scale scores, recovery rate of skin temperature of the hands after the cold stress test, and the Korean version of the WHO Quality of Life Scale, abbreviated version (WHOQOL-BREF).

**Discussion:**

This trial will be the first trial to reflect the newly defined disease range of CHH which was compiled by Korean medicine expert consensus. This study will provide considerable evidence for further large-scale trials and general clinical guidelines for CHH in the Korean medical field.

**Trial registration:**

This study is registered at ClinicalTrials.gov, ID: NCT02645916. Registered on 30 December 2015.

**Electronic supplementary material:**

The online version of this article (doi:10.1186/s13063-017-2002-8) contains supplementary material, which is available to authorized users.

## Background

Cold hypersensitivity in the hands (CHH) is a condition where the individual feels colder in the upper extremities than colder other people in any environment [1, 2]. CHH is not a fatal disease, but can worsen quality of life by interfering with daily activities. According to a Korean genetic etiology and phenotype of CHH study, there are more female CHH sufferers than males with an approximate prevalence ratio of 3:2 [3]. Through reviewing previous CHH-related studies, we found out that studies based on the Asian population were more frequently performed than in the western population. Among the studies, Li et al. reported that the prevalence rates of women with CHH by country are 54.3% in Japan, 25% in Korea, and 20% in China [4].

In conventional medicine, CHH is the representative clinical symptom of Raynaud’s phenomenon (RP) which is classified in the *International Classification of Diseases, 9th edition* (ICD-9) as a peripheral vascular disease. It is thought that an increase in α_2_ sympathetic receptor activity causes the vasoconstriction of blood vessels and central thermoregulatory dysfunction in CHH, but its pathophysiological mechanism is not completely clear [5]. Due to the uncertain pathology and nonstandardized diagnostic and treatment methods of CHH, most conventional physicians prefer to identify CHH by the occurrence of symptoms during the cold stimulation test which is one of the primary diagnostic tests for RP [6]. The general treatment of RP is similar to the allopathic treatments of RP, including vitamin E, calcium-channel blockers, and lifestyle modification, but the efficacy of such treatments is controversial [7].

In Korea, treating CHH with traditional Korean herbal medicine is often preferred to conventional medicine. Because Korean medicine takes a holistic approach to the ailment, it is considered to be a cold-related symptom and is also classified as cold syndrome pattern. Balancing *yin* and *yang* energy in the body with holistic and personalized therapy is one of the advantages of Korean medicine which is more acceptable in Korean society other than symptom-based treatments.

Based on traditional medicine theory, women’s disease is generally caused by cold pathogenic factors [8] which can easily lead to cold-related syndromes such as CHH. Many people consider CHH to be a mild symptom, but it should be treated with careful attention before it worsens and leads to more severe problems.

*Danggui-Sayuk-Ga-Osuyu-Saenggang-tang* (DSGOST) is the one representative traditional Korean herbal medicine formula used to treat CHH since ancient times [9]. The traditional Chinese medicine (TCM) classic, *Shanghanlun* mentions that DSGOST is the appropriate prescription for patients who have had pathogenic internal cold for a long time [10]. It is composed mainly of herbs that can disperse cold and tonify the blood in order to enhance the healing capacity of the body system from cold damage [11]. Nishida et al. [12] found that DSGOST treatment for 8 weeks helped to improve the severity of peripheral coldness and blood flow in female Japanese patients. In our research team, we also found that DSGOST relaxes both the endothelial and vascular smooth muscle cells by inhibiting cold-induced activation of the Ras homolog gene family member A (RhoA) and blocks the endothelin-1-mediated paracrine pathway for the cold response in blood vessels [13]. Although it has a long history of use for CHH, there has been no clinical trial performed to evaluate the potential efficacy of DSGOST in the Korean population. For that reason, through this multicenter, randomized, placebo-controlled trial with participant and investigator blinding, we will examine the feasibility of a full randomized clinical trial of DSGOST on Korean female patients with CHH in order to provide more convincing evidence for the treatment of CHH using herbal medicine.

## Methods

### Trial design

This trial is a pilot, multicenter, double-blind, randomized, parallel-group, two-arm, placebo-controlled clinical trial that will be conducted in the Department of Internal Medicine at Gachon University, Gil Oriental Medical Hospital, Dongguk University, Ilsan Oriental Hospital, Sangji University Oriental Medical Centre, Semyung University Second Affiliated Oriental Medical Hospital at Chungju, and the Department of Gynecology, Kyung Hee University Oriental Medical Center, Kyung Hee University Oriental Medicine Hospital at Gangdong in Korea.

**Fig. 1:**
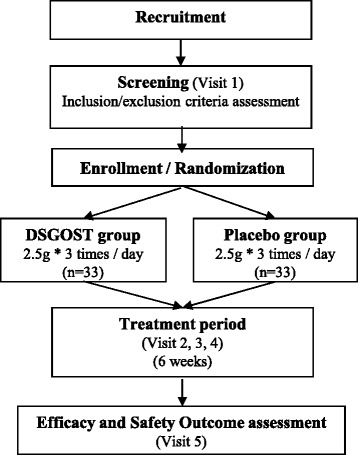
Flow chart of the trial process

We will recruit 66 participants in total. Once the participant decides to take part in this trial, there will be at least a 2-week run-in period prior to randomization. After randomization, each participant will be treated for 6 weeks and visit the clinic once every 2 weeks. The total trial duration will be 8 weeks. The flow chart of the trial is shown in Fig. 1.

### Randomization

Eligible participants who consent to participate will be randomly assigned to either the treatment (DSGOST) or the placebo group with an allocation ratio of 1:1. Assignment will be done with the web-based randomization system developed by the contract research organization (CRO), the Institute of Safety and Effectiveness Evaluation for Korean Medicine (ISEE). The random sequence will be generated by an independent professional statistician from the CRO using the SAS 6.1 program and stratified by hospital using random block sizes of 2 and 4.

### Blinding

Once the participant is randomly allocated to a group, neither the investigator nor the participant will know which type of the drug the participant receives until the end of the trial. The trial drugs will be manufactured in packages that are indistinguishable in appearance and then distributed by independent research pharmacists or assistants who are also blinded to the randomization, in each hospital. When voluntary withdrawals or urgent medical situations, such as severe adverse events (SAEs), are encountered, the principal researcher of the hospital will inform the sponsor and the CRO immediately to handle unblinding according to standard operating procedures (SOPs).

## Participants

### Inclusion criteria

This study will enroll a total of 66 patients who have a complaint of CHH and who are eligible according to the following criteria:

1. Female subjects aged 19 to 59 years who have a complaint of CHH.
2. Patients must have at least one of the following symptoms:
  - Symptoms of CHH at a temperature at which most individuals feel no cold
  - Symptoms of extremely cold hands upon cold temperature exposure
  - Those who, upon return to a warmer environment, continue to have symptoms of cold hands
3. A 4-cm or greater Visual Analog Scale (VAS) CHH score
4. Those in whom the thermal difference between the palm (PC8) and the upper arm (LU4) is higher than 0.3 °C
5. The ability to comply with all study-related procedures, medications, and evaluations
6. The signing of a written Informed Consent Form

### Exclusion criteria

The exclusion criteria are as follows:

1. Patients who have taken calcium antagonists or beta-blockers for the purpose of treating CHH
2. Those who have gangrene or ulceration on one or more fingers
3. Those who have been diagnosed with hypothyroidism or are currently taking thyroid medications
4. Those who have been diagnosed with an autoimmune disease or have a positive antinuclear antibody (ANA) test result
5. Those who have been diagnosed with carpal tunnel syndrome or have positive Tinel’s and Phalen’s tests
6. Those who have been diagnosed with cervical disc herniation or have a malignant tumor
7. Those who have been diagnosed with diabetes
8. Those who are currently medicated with drugs that may affect CHH symptoms such as anticoagulants
9. Those who have a moderate level of liver dysfunction (each of AST and ALT greater than 100 IU/L) or kidney dysfunction (creatinine greater than 2.0 mg/dL)
10. Those who currently receive psychological or psychiatric treatment due to any mental illness, such as behavior disorder, depression, anxiety, neurosis, schizophrenia, or any other serious mental illness
11. Those who have been diagnosed with moderate anemia or hematological disorders (adult nonpregnant women hemoglobin (Hgb) level less than 7 g/dL, hematocrit (Hct) less than 26%, white blood cell (WBC) level greater than 11,000/mm^3^)
12. Those whose systolic blood pressure (SBP) is greater than 180 mmHg or whose diastolic blood pressure (DBP) is greater than 100 mmHg, based on the average of at least two measurements
13. Those with an arrhythmia that shows on an electrocardiogram (ECG) or who have been diagnosed with heart diseases such as ischemic heart disease
14. Those who are addicted to alcohol or drugs
15. Those who are pregnant (positive urine-HCG) or lactating or may become pregnant
16. Those who are currently participating in other clinical trials
17. Those who are unable to understand and speak Korean
18. Those who are judged to be inappropriate for the clinical study by the researchers such as refusal to comply, forgetting visits, lack of comprehension, etc.

### Withdrawals

Voluntary participation and informed consent are prerequisites for this trial before enrollment. All participants have the right to refuse to continue the study at any time for any reason. Reasons for withdrawal will be documented in Case Report Forms (CRFs) and data will be analyzed using the intention-to-treat (ITT) principle.

### Recruitment

Participant recruitment will take place in six hospitals of Korean medicine in various Korean cities. Each institution will advertise the trial by posting recruitment posters on the hospital website or bulletin board. If necessary, the local community newspaper advertising will also be considered.

### Interventions

After enrollment, participants will take 9 g of DSGOST or placebo drug per day, for 6 weeks. Both groups will take the assigned drugs orally with warm water three times a day, at least 30 min after a meal.

The investigational drug, DSGOST is a well-known traditional herbal formula for treating cold syndrome in traditional medicine. In this trial, we will use DSGOST granulated extract (manufactured by Hanpoong Pharm and Foods Co., Ltd. (HP)) for convenience of use. It contains 1.33 g of *Zingiberis Rhizoma*, 1.67 g of *Zizyphi Jujubae Fructus*, 1.00 g of *Cinnamomi Ramulus*, 1.00 g of *Paeoniae Lactiflorae Radix*, 1.00 g of *Angelicae Gigantis Radix*, 0.67 g of *Asari Radix*, 0.33 g of *Evokiae Fructus*, 1.00 g of *Akebiae Lingnum*, and 0.67 g of *Glycyrrhizae Radix*.

Placebo drug will also be prepared and will pass several quality assurance tests. It will have a relatively similar color, shape, weight, odor, and taste to DSGOST so that participants will not notice whether they are given the real drug or placebo. The daily dose of placebo granules contain 5.1 g of lactose, 3.0 g of cornstarch, 0.3 g of citric acid, 0.3 g of pigments, and 0.3 g of *Ssanghwa* herbal flavor.

### Outcomes

**Fig. 2:**
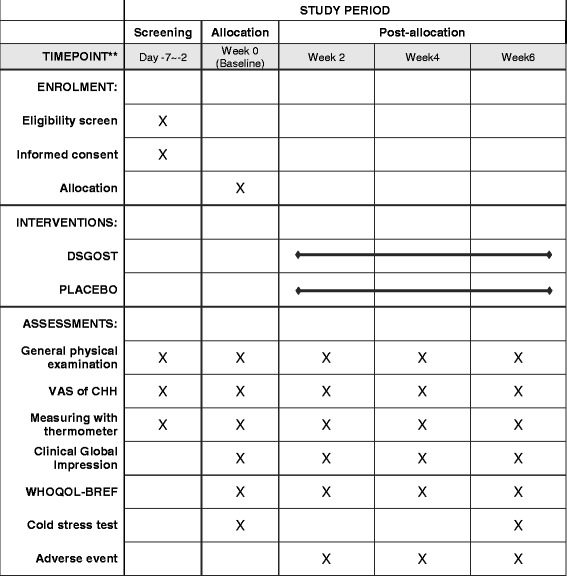
Study schedule of the *Danggui-Sayuk-Ga-Osuyu-Saenggang-tang* (DSGOST) clinical trial

In this study, the primary outcome will be measured using Visual Analogue Scale (VAS) scores of cold hypersensitivity in the hands. Secondary outcomes will include changes in skin temperature of the hands, Clinical Global Impressions (CGI) scale scores, recovery rate of skin temperature of the hands after the cold stress test, and the Korean version of the World Health Organization (WHO) Quality of Life Scale, abbreviated version (WHOQOL-BREF). The schedule of treatment and endpoint measurements are listed in Fig. 2.

## Primary outcome measure

### Visual Analogue Scale (VAS)

A 100-mm VAS scale will be used to measure the severity of CHH on a 10-point scale ranging from 0 (no coldness) to 10 (most severe coldness). The change of CHH VAS score between groups will be recorded at every visit throughout the entire trial.

## Secondary outcome measure

### Body skin temperature (BT)

Patients will be forbidden from consuming caffeinated drinks, alcohol, tobacco, undergoing vigorous exercise, and taking hot showers for at least 2 h before BT measurement. After a 15-min relaxation period in the exam room at 24 ± 1 °C, BT of both palms (PC9) and anterior upper arms (LU4) will be measured by thermometer (Testo 835-T1, Lenzkirch, Germany). The specific acupuncture point location will be determined by experts according to SOPs and performed at every visit.

### Clinical Global Impressions (CGI)

The Clinical Global Impression (CGI) rating scale is a 7-point scale developed to estimate treatment response and efficacy in patients with mental disorders and it is administered by a physician. The CGI is composed of two scales, one for evaluating the severity of disease (CGI-S) and one for evaluating symptom improvement (CGI-I) [14]. In this study, considering the subjective nature of CHH, we decided to use the CGI scale as a secondary outcome measure to support the accuracy of the primary outcome measure. It will be measured during visits 2, 3, 4, and 5.

### Cold stress test (CST)

The CST is normally used for diagnosing vascular disorders such as hand-arm vibration syndrome and Raynaud’s phenomenon. Its procedure is not standardized [15], so we adapted the measurement method of the CST from a previous study by Park et al. [16]. The CST will be performed at visits 2 and 5.

Patients will sit quietly for 20 min in the exam room (24 ± 1 °C) before the procedure. After the relaxation period, the temperature of both palms will be measured using an infrared thermometer (Testo 835-T1, Lenzkirch, Germany), then both hands will be immersed into a container of iced water (20 °C) for 30 s. After cold water immersion, the hands will be dried thoroughly and the temperature measured by thermometer at the same points that were measured before immersion. The skin temperature 6 min after the CST will also be measured. The recovery rate (*RR*) of the skin temperature between, before, and after the CST, and 6 min after the CST, will be computed as follows:

$$ R R=\left({T}_6-{T}_0\right)/\left({T}_{base}-{T}_0\right)\times 100\left(\%\right) $$

*T*_base_: The skin temperature at baseline

*T*_0_: The skin temperature measured by using a thermometer immediately after the CST

*T*_6_: The skin temperature measured by using a thermometer at 6 min after the CST

### WHOQOL-BREF

To estimate quality of life scores, the WHOQOL-BREF, invented by Min et al. [17], will be used. The WHOQOL-BREF is a 26-item generic questionnaire containing five domains: general quality of life, physical health, psychological health, social relationships, and environmental health. It will be checked at visits 2, 3, 4, and 5.

### Safety assessment

The safety assessment, including physical examination, renal function tests (blood urea nitrogen (BUN), creatinine), and liver function tests (ALT, AST, and γ-GT), will be conducted at visits 2 and 5. Vital signs will be checked at every visit.

### Adverse event reporting

All participants will be required to report any adverse events (AE) that occur during the trial at every visit. Each AE must be recorded in the CRF by the site investigator. All AEs will be assessed for causality. If serious adverse events (SAEs) occur, the Institutional Review Board (IRB) and principle investigator will be notified as soon as possible. After receiving an SAE report, the site investigator will decide whether the participant must withdraw or whether they may continue to participate in the trial.

### Sample size calculation

This clinical trial is a pilot study to examine the feasibility of a full randomized clinical trial of DSGOST and determines the effect size for further large-scale studies. We required a sample size of 66 based on requiring data from 25 participants per arm to calculate estimates of treatment effect to inform the power calculation for a main study. We accounted for a 20% dropout rate based on a similar study [16].

### Statistical analysis

An independent statistics expert will perform the statistical analysis in a blind manner. Efficacy analysis will be conducted for both intent-to-treat (ITT) and per-protocol (PP) analysis. For the primary outcome measurement, the mean difference of VAS score between the experimental group and the placebo group will be analyzed using independent sample *t* tests or Mann-Whitney *U* tests for continuous data and chi-square or Fisher’s exact tests for categorical data. The repeated measures analysis of variance (ANOVA) will be used to assess the presence of any significant difference in the mean VAS, BT, CGI, and WHOQOL-BREF scores between groups at different time points (baseline, visits 3, 4, and 5) as a secondary outcome. Regression analyses also be used examine the effect of DSGOST on outcomes for adjusting the baseline data and study sites. Comparison of means of continuous variables (i.e., VAS, BT, CGI, and WHOQOL-BREF scores) will be expressed as differences in means with 95% confidence intervals (CIs).

Missing data will be adjusted using the last-observation-carried-forward (LOCF) imputation method. Statistical analyses will be performed using the SPSS 23.0 program. The statistical level of significance will be established at *P* < 0.05.

### Data and safety monitoring

The ISEE will monitor the clinical trial. The monitoring will begin when the first participants complete the required number of visits. All the investigational institutions will be monitored based on the SOPs while this trial is in process. Auditing is not scheduled for the present study. To improve data quality, range checks for data values and double data entry will be done. Any other committee, such as a coordinating center, a steering committee, an endpoint adjudication committee, etc., will not be applicable in the present study.

### Ethical consideration

This trial was approved by the Institutional Review Board (IRB) of six different hospitals (Gachon University Gil Oriental Medical Hospital: 15-103, Kyung Hee University Oriental Medical Center: KOMCIRB-150818-HR-032, Kyung Hee University Oriental Medicine Hospital at Gangdong: KHNMCOH2015-09-002-002, Dongguk University Ilsan Oriental Hospital: 2015-09, Sangji University Oriental Medical Centre: SJIRB-DRUG-15-002, and Semyung University Second Affiliated Oriental Medical Hospital at Chungju: 1511-08). This study will abide by the amended Declaration of Helsinki and the regulations of the “Good Clinical Practice” principles of the Korea Food and Drug Administration (KFDA). The current protocol version is 1.5 and it has been developed according to the Standard Protocol Items: Recommendations for Interventional Trials (SPIRIT) Statement (see Additional file 1). All items from the World Health Organization Trial Registration Data Set have been drawn. All participants will receive sufficient explanation and time to determine trial participation. Written informed consent will be obtained from all participants before beginning any trial procedures.

## Discussion

CHH is a subjective symptom that is commonly seen in women in Korea. In conventional medicine (CM), CHH is categorized as an associated symptom of vascular diseases such as RP and hand-arm vibration syndrome. In contrast, traditional Korean medicine (TKM) defines CHH as a disease entity in itself which is a key factor for the final diagnosis of cold syndrome pattern. According to one Japanese survey, approximately 60% of the Japanese population suffer from cold syndrome, especially extremity coldness [18]. Facing health care issues, the Japanese government approved insurance coverage of *Kampo* treatment for CHH [19]. However, even though there have been increasing numbers of people experiencing CHH in Korea, policymakers have not yet allowed for broader TKM health insurance coverage or better development of a TKM welfare program.

In designing this trial, we reviewed previous studies by searching various databases (EMBASE, PubMed, CNKI, Oasis). We found several studies on CHH in the traditional medicine field, but its diagnostic criteria and terminology were varied. In addition, most of the studies focused on the occurrence of symptoms upon exposure to cold temperatures.

To determine new diagnostic criteria for CHH in TKM, we organized an Advisory Committee of Experts (ACE) consisting of 10 medical experts, each with more than 10 years of clinical experience, as well as experience treating CHH. The Delphi survey method was used for making accurate decisions. As a result, the collated determination of the ACE was that most Korean women with CHH complain of coldness in the hands at room temperature as well as from cold temperature exposure [1]. Thus, the ACE considered expanding the range of diagnostic criteria to include patients with symptom occurrence both at room temperature and at cold temperatures. Thus, it will be significant to perform a clinical trial using a broader perspective of CHH in TKM. It will also help to define the characteristics of CHH in the Korean population.

Although a few previous studies used infrared thermography to measure the skin temperature with scientific accuracy, this may not reflect the severity of patients’ actual self-report of discomfort due to the limitation of objective measurement. In this exploratory trial, we instead decided to use a VAS score as the primary outcome measure for the severity of CHH changes between baseline (visit 2) and after treatment (visit 5).

In this trial, the CST will be applied to evaluate functional recovery after cold stress in patients with CHH. It is a well-known test for diagnosing RP [20] and hand-arm vibration syndrome [21] and has a high sensitivity and specificity [22]. However, the CST procedure is not yet standardized. In general, earlier studies used a varied temperature from 0 °C to 15 °C and an immersion time from 1 to 20 min [15]. In this trial, the ACE recommended a water temperature of 20 °C and an immersion time of 1 min in order to minimize uncomfortable pain. This was based upon the clinical experience of ACE members and the results of previous validation tests.

There are a few limitations in this trial. Firstly, due to the relationship between cold environment and target disease, the results of this study might be affected by environmental temperature. However, we try to select well-performing study sites by feasibility assessment of equipment and facilities required to successfully conduct the trial such as the availability of room with well-controlled temperature, for reducing any potential bias. We also consider the differences of daily temperature between cities in Korea, so we will recruit the participants at six hospitals in five different regions of Korea to explore the characteristics of CHH differences within the Korean peninsula. Secondly, due to the small research grant for the pilot study, we will not be able to completely exclude the presence of RP. However, an ANA test will be performed to rule out any autoimmune disorders, instead of a primary test for RP.

Our team plans to conduct further large-scale clinical trials using several Korean medicine extracts for CHH in the near future. The results of this study will provide valuable information, such as methodological problems, specified outcome variables, regional characteristics of CHH, and statistical power for sample size calculation, for future large-scale trials. Furthermore, it will provide supporting evidence for the future development of general clinical guidelines for CHH in the Korean medicine field.

### Trial status

Recruitment is ongoing from December 2015.

## Additional file

Additional file 1: SPIRIT Checklist. Recommendations for scientific, ethical, and administrative elements that should be addressed in a clinical trial protocol. (PDF 39 kb)

## Abbreviations

ANA: Anti-nuclear antibody
BP: Blood pressure
BUN: Blood urea nitrogen
CHH: Cold hypersensitivity in the hands
Cr: Creatinine
CRO: Clinical Research Organization
ECG: Electrocardiography
hCG: Human chorionic gonadotropin
Hct: Hematocrit
Hgb: Hemoglobin
IRB: Institutional Review Board
ISEE: Institute of Safety and Effectiveness Evaluation for Korean Medicine
ITT: Intention-to-treat
LOCF: Last observation carried forward
PP: Per-protocol
RBC: Red blood cell
SAE: Severe adverse event
SOP: Standard operation procedure
VAS: Visual Analogue Scale
WBC: White blood cell
WHOQOL-BREF: World Health Organization Quality of Life Scale brief version
